# Undifferentiated sarcoma originating from the mitral valve: a case report

**DOI:** 10.1186/s13019-016-0447-6

**Published:** 2016-05-14

**Authors:** Haibin Zhang, Weitie Wang, Dan Li, Zhicheng Zhu, Tiance Wang, Rihao Xu, Kexiang Liu

**Affiliations:** Department of Cardiovascular Surgery, 2nd Hospital of Bethune, Jilin University, Changchun, Jilin China

**Keywords:** Cardiac tumors, Sarcoma, Pathology, Cardiac surgery

## Abstract

**Background:**

Primary cardiac sarcomas are quite uncommon. Among them, sarcomas originating from the mitral valve are exceedingly rare. They are often misdiagnosed due to non-specific symptoms. The prognosis of cardiac sarcomas remains poor, and finding a more effective therapy poses a big challenge for the doctors. Currently, complete surgical resection of the tumor is still the most popular treatment in cases that without metastases.

**Case presentation:**

A 38-year-old female was transported to our department with persistent cough and chest pain. The operation of mitral valve replacement was scheduled after a provisional diagnosis of serious mitral stenosis. During the operation, we found that a large polypoid mass infiltrating mainly the mitral valve and part of the left atria. We removed the tumor as completely as possible and replaced the mitral valve. Histology confirmed the diagnosis of undifferentiated sarcoma after operation. The patient was asymptomatic with no recurrence at 3-month follow-up.

**Conclusion:**

Primary cardiac sarcomas is a rare and the prognosis is poor. The sarcomas is often found by chance during the operation. Surgical operation need to be carried out directly.

## Background

Primary cardiac tumors are rare with approximately 25 % of them are malignant. Sarcomas account for 75 % of the cardiac malignant neoplasms [1]. Cardiac sarcomas include angiosarcoma which is the most common type, sarcoma with myofibroblastic differentiation, synovial sarcoma and rhabdomyosarcoma. Cardiac sarcomas are often asymptomatic until hemodynamic is influenced by tumor. The ages of presentation are more likely to be less than 65 year-old [2] and the mean age is around 40 years [3]. They are a rare entity in infants and children and equally prevalent in both genders.

Due to rapidly proliferating and distant metastases, the prognosis of sarcomas is poor. The median survival of patients without metastasis is 15 months, in contrast to 5 months for those with metastasis [4]. We present a rare case of sarcoma originating from the mitral valve, which was successfully treated by surgical management.

## Case presentation

A 38-year-old female presented with 4 months history of persistent cough with shortness of breath and chest pain. Laboratory tests and physical examination were unremarkable. Chest × ray suggested the pulmonary congestion. Electrocardiography revealed sinus rhythm. The morphological changes of the mitral valve revealed by echocardiography included leaflet and subvalvular apparatus thickening, shortened chordae tendineae, and commissural fusion. The tricuspid valve was intact and right ventricular and atrial sizes were normal under echocardiography.

**Fig. 1:**
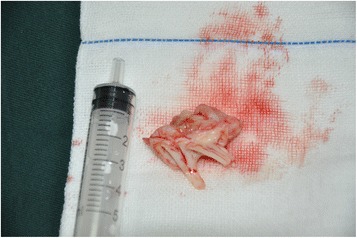
Intraoperative photograph of the resected sarcoma: polypoid mass infiltrated the entire anterior mitral leaflet

The operation of mitral valve replacement was scheduled after a provisional diagnosis of serious mitral stenosis. A traditional median longitudinal sternotomy was implemented. After full heparinization, cardiopulmonary bypass (CPB) was routinely applied, and cold cardioplegic solution was instilled via the aortic root after cross clamping the aortic. Upon the opening of the left atrium by longitudinal incision, we observed unexpectedly that a large polypoid mass infiltrated the entire anterior mitral leaflet and part of posterior mitral leaflet, resulting in a 0.5 cm^2^ orifice area. In addition, part of the left atrium was also involved with the same lesion. According to the character of the mass, we considered it to be heart sarcoma. After a complete resection of the leaflets and chordae tendineae and a partial resection of papillary muscle [Fig. 1], we excised part of the left atrium that was infiltrated by the tumor as completely as possible without destroying the full-thickness of left atrium. Lectrocoagulation electrotome setting in 20 J burned the remains of the mitral valve and part of the left atrium. Mechanical heart valve (size 27) replacement was executed in the end. As expected, the heart was beating in sinus rhythm. After careful hemostasis and closing the wound in layers, the patient was carefully transferred to the intensive care unit (ICU) in stable condition. The patient made an uneventful recovery with all pre-operative symptoms resolved and was discharged home on postoperative day 9. She was doing well without signs of recurrence during the 3 months postoperative follow up.

**Fig. 2:**
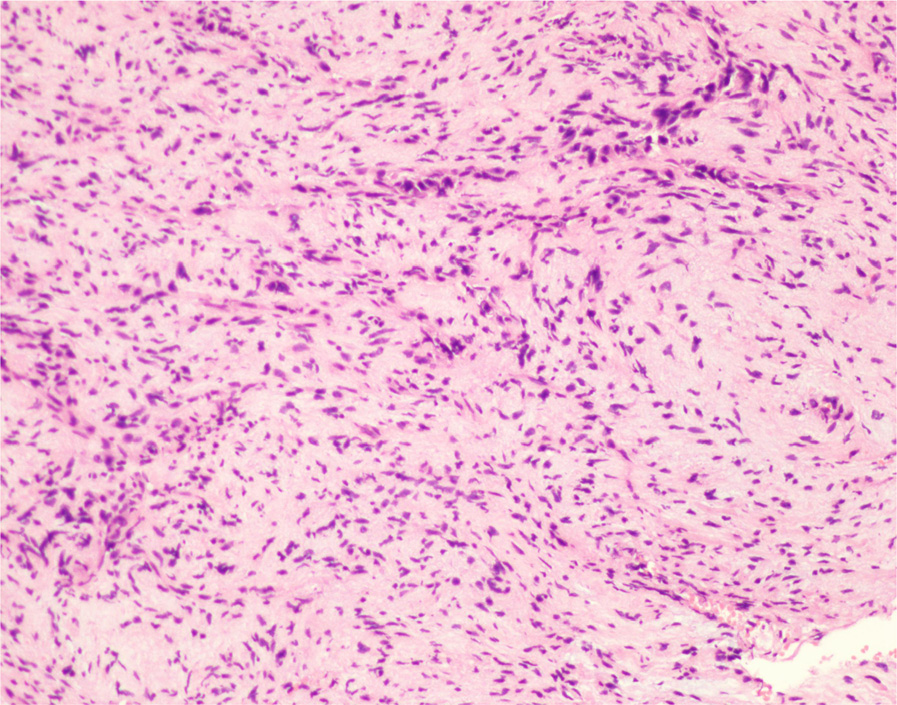
Histology showing appearance of sarcoma: tumour cells arranged in characteristic storiform, interlacing fascicular and diffuse patterns

On immunohistochemical examination, the tumor cells stained positive for CD34, CD31, vimentin and WT1 but negative for CK(AE1/AE3), Desmin, Calretinin, CK5/6, S-100 and HMB45. Histology confirmed the diagnosis of undifferentiated sarcoma [Fig. 2].

## Discussion

Upon reviewing of current literature, we discovered that primary sarcomas were occasionally found in the left atrium, left ventricle and right atrium with undifferentiated sarcomas usually found in left atrium. However, the tumor originating from mitral valve and infiltrating the left atrium has not been reported. In this study, we report a case with primary sarcoma infiltrating mainly mitral valve, suggesting that the origin of the tumor was most likely the mitral valve. Considering of the possible origin of the sarcoma, we focused on dealing with the primary lesion during the surgical treatment. Before replacement of mitral valve, electrotome burning of the remains of the mitral valve and part of the left atrium infiltrated by the sarcoma was implemented to avoid recurrence.

Although the true diagnosis of primary cardiac sarcoma is often made as an unexpected finding during the operation, the development of non-invasive cardiac imaging such as Transthoracic Echocardiography (TTE) and Transesophageal Echocardiography (TEE) has vital value in the preoperative assessment of cardiac sarcoma [5]. However, these pictures sometimes were hard to interpret properly as shown in our case and others. CT and MRI may be applied as complementary to echocardiography to facilitate the identification of the tumors and adjacent invasions.

Standard treatments for primary cardiac sarcomas have not been defined yet due to their low incidence. There are currently many treatment options such as surgical excision, radiotherapy, chemotherapy, heart transplantation and complex treatment. Among these managements, completely surgical excision is the most popular therapy that has shown to prolong the survival [2]. The effect of adjuvant chemotherapy remains uncertainty despite that some reports have shown improvement in long-term survival. In consideration of the locally aggressive nature of cardiac sarcomas, heart transplantation, an effective approach, has been suggested as a new method to cure some special cases. However, the fact that many patients are dying of transplant related complications makes some people suggest that cardiac transplantation may restrict the improvement in survival [6, 7].

## Conclusions

The current study reported a rare case of primary cardiac sarcoma that possibly originating from mitral valve and infiltrating part of left atrium. The tumor was completely excised through surgery and the patient was recovered without complications. Since the tumor infiltrated both the mitral valve and part of left atrium, the true origin of the tumor is still a puzzle, which is the limitation of this study.

## Consent

Written informed consent was obtained from the patient for publication of this Case report and any accompanying images. A copy of the written consent is available for review by the Editor-in-Chief of this journal.
